# Grape Seed Extract as an Adjuvant Therapy for Huntington Disease; A Narrative Review of Biological Plausibility and Potential Clinical Outcomes

**DOI:** 10.3390/molecules31142402

**Published:** 2026-07-08

**Authors:** Carolyn DeBoth, Jessie Kasper, Casey McDonald, David M. Duriancik

**Affiliations:** College of Medicine, Lake Erie College of Osteopathic Medicine, 1858 W. Grandview Blvd., Erie, PA 16509, USA

**Keywords:** grape seed extract, polyphenols, Huntington disease

## Abstract

Huntington disease (HD) is a debilitating, genetic disorder with a prevalence of 2.7 per 100,000 people. It is neurodegenerative, leading to cognitive, behavioral, and motor symptoms from neuronal loss within the striatum of the basal ganglia and cortex. Currently, the treatments involve symptomatic management, instead of treating the pathophysiology of the disease. Grape seed extract (GSE) is a complex mixture of polyphenols, proteins, and lipids with antioxidant and anti-inflammatory properties. This literature review examines the possibility of using GSE as a potential adjunctive therapy for HD. Preclinical studies have shown a neuroprotective effect through biologically plausible mechanisms. Clinical research has shown that GSE works on redox and inflammatory pathways related to the pathogenesis of HD. Although there are not many clinical trials on GSE in HD patients directly, the overlap of mechanisms behind both GSE and HD and the favorable side effect profile make GSE a potential adjunctive therapy. Targeted clinical investigation is warranted to determine the full therapeutic potential of GSE.

## 1. Introduction/Background

Huntington disease (HD) is an autosomal dominant disorder due to a gain of function mutation that has a worldwide prevalence of 2.7 per 100,000 people [1,2]. HD is typically diagnosed by confirmed family history or a positive genetic test with the onset of motor disturbances categorized by the Unified HD Rating Scale total motor score and diagnostic confidence score. These scales analyze eye movements, speech, alternating hand movements, dystonia, chorea, and gait to gain an understanding of the stage of the disease [3]. Symptoms of HD include severe cognitive and personality changes along with deficits in coordination and motor skills leading to immobility. Specific symptoms include chorea, loss of coordination, weight loss, skeletal muscle wasting, cardiac failure, depression, psychosis, and obsessive–compulsive disorder [4].

The signs and symptoms of HD are due to neuronal loss in the striatal portion of the basal ganglia and cortex, specifically the putamen and caudate [1,2]. With disease progression, cortical ribbon thinning moves from posterior to anterior [4]. This leads to changes in motor symptoms throughout the course of the disease, with the beginning of the disease presenting with chorea, and then as it progresses, HD moves into the hypokinetic phase with bradykinesia, dystonia, and balance and gait disturbances [5,6]. GABA is also decreased in the striatum, palladium, and substantial nigra [7]. The symptoms of HD are likely dependent on the region of the brain that is affected by the cell death and neurodegeneration occurring. The pathogenesis of HD is continuing to be studied with multiple proposed mechanisms, including neuronal aggregates with inclusions of the mutant HTT genes causing dysregulation in the ubiquitin–proteosome pathway, transcriptional dysregulation, excitotoxicity from an increase in the amount of glutamate, mitochondrial dysfunction with impaired energy metabolism, neuroinflammation, and alterations in axonal transport and synaptic dysfunction [1].

The management of HD consists of symptomatic management with no cure found yet. For movement disorders like chorea, tetrabenazine is the first-line treatment. This medication reversibly inhibits vesicular monoamine transporter 2 (VMAT2), decreasing the amount of dopamine present in the central nervous system (CNS) and leading to many adverse effects including depression, insomnia, akathisia, and somnolence when initiated. Due to the emotional and behavioral effects HD has on patients, it is important to monitor them closely when adding this medication to their regimen. Other medications with a similar mechanism of action include dopamine antagonists, which have helped with treating motor and psychiatric symptoms but also have adverse effects such as akathisia and tardive dyskinesia. Amantadine is an NMDA receptor antagonist which helps decrease chorea when prescribed in a higher dose. Parkinson disease medications, such as levodopa, dopamine agonists, and amantadine, may help patients with HD that display parkinsonian features. When patients have focal dystonias, botulinum toxin is beneficial. Psychiatric symptoms should be addressed with non-pharmacological treatments such as counseling since antidepressant medications have not been shown to be effective. Treatments are continuing to evolve with HD including stem cell therapies, gene therapies, and clinical trials on AMT-130, which targets rAAVS-miHTT, lowering the levels of mHTT, and ALN-HTT02, which leads to RNA interference, targeting exon 1 of HTT mRNA [8,9,10]. However, no cures have been confirmed yet, so possible adjunctive therapies should continue to be investigated.

Brain deterioration in HD is due to a cytosine, adenine, and guanine (CAG) trinucleotide repeat within the Huntingtin (HTT) gene on chromosome 4p16.3 [1]. These repeats are not stable as they increase in size, leading to the phenomenon of anticipation [4]. The average age of onset of HD is around 40, although this can vary due to genetic anticipation, and this disease affects males and females at an equal frequency [2]. Once mutated, the HTT gene can aggregate and cause transcriptional dysregulation, proteasomal dysfunction, induction of autophagy, release of calcium from intracellular stores, mitochondrial failure, apoptosis, and excitotoxicity in extrasynaptic NMD receptors [2,11,12]. The number of repeats in HTT affects both the age of onset and the presentation of the disease. For patients with greater than or equal to 42 repeats, there is complete penetration of the disease, but for those with 36–41 repeats, some lead a normal lifespan with signs of HD.

A key limitation in the treatment of HD is that there is not a cure. Current therapies are used to improve quality of life and decrease symptoms but not impact lifespan or target the underlying pathophysiology of the disease. Recently, polyphenols have been shown to have neuroprotective properties which may help with increasing lifespan and decreasing motor symptoms in patients with HD [13]. Grape seed extract (GSE) is considered a cost-effective, polyphenolic-rich, and safe therapy with mild adverse effects [14]. For this reason, GSE should be addressed as a possible adjunctive therapy to help the management of HD and address the pathology behind the disease.

In this review, we discuss the composition of GSE and the biological activity of each component, such as anti-inflammatory, antioxidant, and antimitochondrial effects, with its role in reducing signs and symptoms of HD. We summarize the biological plausibility as well as the safety profile of GSE. We show the evidence that is currently applicable to the use of GSE in HD, while urging for more prospective clinical trials testing the use of GSE as an adjuvant therapy in patients with HD.

## 2. Methods

To identify studies evaluating GSE, its polyphenolic constituents, and their potential neuroprotective roles in HD, we conducted a comprehensive literature review. We utilized PubMed and Phenol Explorer as our primary databases. The search strategy included the keywords “Huntington Disease”, “Grape Seed Extract”, Proanthocyanidins”, and “Polyphenols”. We included studies that assessed GSE or its metabolites in relation to neurodegeneration-relevant mechanisms, such as oxidative stress, neuroinflammation, mitochondrial dysfunction, or blood–brain barrier penetration. Studies unrelated to GSE and neurodegenerative outcomes were excluded.

## 3. Grape Seed Extract: Composition and Compounds

Grapes are one of the most popularly grown fruits worldwide, with major producers including China, Italy, France, and the USA [15]. They are typically used in the winemaking process, where the crushing of 1 kg of grapes produces 0.3 kg of solid byproducts called grape marc. Grape marc typically consists of 25% grape stalks, 25% seeds, and 50% skins [16]. Grape seeds can be isolated from grape marc and are composed of approximately 13–19% oil, including both polyphenols and fatty acids, 11% protein, and 60–70% fiber [17]. However, the exact composition of grape seeds can vary and depend on the condition in which they are grown. Drought conditions were shown to decrease total phenolic compounds in grapevines and the antioxidant properties long-term along with metabolism to conserve energy [18]. However, moderate water stress conditions have increased the amount of phenolic compounds in grape seeds, showing that phenolic compounds can become a protective mechanism for the seeds, but there is a maximum level that they reach [19]. This same theory was also shown with stress caused by temperature and UV radiation, where the phenolic acid production hit a maximum in protection when the stressor became severe [20]. Recently, grape seed oil or extract has become an increasingly desired product on the market specifically for Australia, Korea, Japan, and the US [21]. There are two techniques to produce GSE from grape marc: organic solvent or mechanical methods. The organic solvent method involves solvent recovery by distillation to produce a favorable extraction yield, however, this is the less desirable method because it contains traces of residual solvent [16]. This residual solvent can be a risk as the oral effects of the solvents used, such as ethanol, methanol, hexane, and petroleum ether, are not well studied, and risks could include both neurological and developmental effects [22]. The mechanical method of extraction involves filtration and is more attractive because of its higher product quality even though there is a lower total yield [16].

Once formed, GSE consists of many key bioactive compounds. Phenols compose the majority of the bioactive component of GSE, with a phenolic percent composition of 5–8% in total seed weight [23]. Its major phenolic compounds are flavan-3-ols (proanthocyanidins), flavonols, hydroxycinnamic acids, and hydroxybenzoic acids [24]. The specific flavonoids in GSE can vary widely by variety, however, they typically consist of gallic acid, catechin, epicatechin, gallocatechin, epigallocatechin, epicatechin 3-O-gallate [23]. The specific concentration of each flavonoid is variable, with, for example, catechin and epicatechin concentrations ranging from 12–358 mg/100 g and 96–421 mg/100 g dry matter respectively across different grape varieties [25]. GSE also contains antioxidant vitamins, specifically vitamins C and E [26]. GSE was also found to contain both saturated and unsaturated fatty acids, with the most abundant being polyunsaturated fatty acids (85–90%) of which the majority (65%) was found to be linoleic acid [27,28]. GSE is associated with multiple enzymatic and non-enzymatic antioxidant compounds through the antioxidant proanthocyanidins [29]. Despite GSE being a heterogeneous mixture, each of the bioactive components described above has the ability to independently exert specific and significant metabolic functions that protect against further cellular damage due to HD while the combined effects found in GSE may provide additive or synergistic benefits.

## 4. Grape Seed Extract Mechanisms on Huntington’s Disease as an Adjunctive Therapy

**Figure 1:**
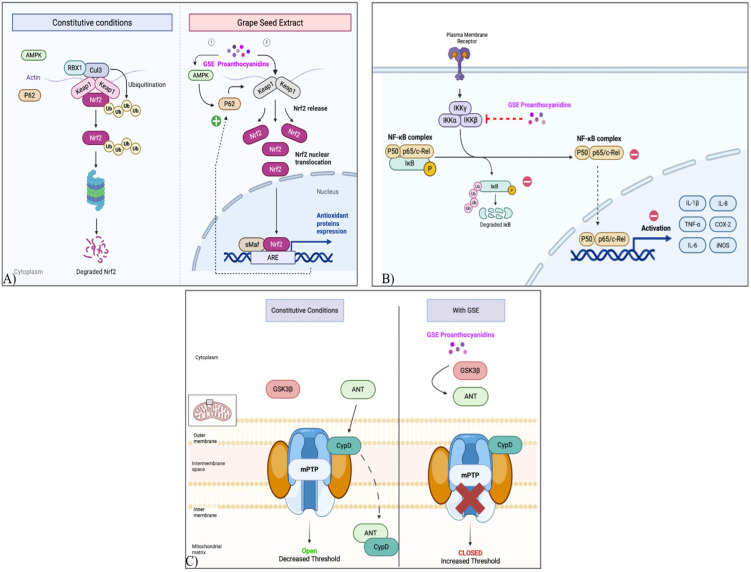
Grape Seed Extract Molecular Mechanisms. (**A**) Antioxidant Effect of GSE on Nrf2 Pathway. Two primary molecular mechanisms. Under constitutive conditions, Keap1 is bound to Nrf2 while Cul3 binds and signals Nrf2 for ubiquitination and therefore proteasomal degradation. With the addition of GSE, GSE frees Keap1, therefore stabilizing Nrf2. Nrf2 is then able to enter the nucleus and bind Maf, which binds antioxidant elements to encode antioxidant and detoxifying enzymes. Figure designed in BioRender with template adapted from Samara Ona, Science Designer. (**B**) Anti-inflammatory Effect of GSE on NF-κB Pathway. GSE crosses the plasma membrane and enters the cell cytoplasm. GSE then targets the IKKα/β complexes for inhibition. The inhibition of IKKα/β complex prevents IKβ from binding P50 and p65/c-Rel, therefore preventing P50 and p65/c-Rel from entering the nucleus. This prevents the transcription of proinflammatory cytokines including TNF-α, IL-1β, IL-6, IL-8, COX-2, and iNOS, thus decreasing the inflammatory state. Figure designed in BioRender with template adapted from Akiko Iwasaki, Sterling Professor at Yale University School of Medicine. (**C**) GSE Effect on Mitochondrial Permeability Transition Pore Threshold of Apoptotic Protein Release. In the absence of GSE, ANT is freely available to bind CypD on the mPTP, causing dissociation of CpyD from the mPTP and causing a difference in membrane potential, which then increases permeability through the pore. This decreased threshold allows increased transport of calcium ions and ROS to initiate the apoptosis pathway. In the presence of GSE, there is increased phosphorylation of GSK-3β. This allows GSK-3β to bind ANT and prevents ANT from binding CypD. CypD remaining bound to the mPTP maintains the mitochondrial membrane potential and increases the permeability threshold. This prevents the apoptotic cascade from beginning. Figure created in BioRender by Carolyn DeBoth, Jessie Kasper, and Casey McDonald. (2026). https://biorender.com/.

GSE has many beneficial properties for HD through its biochemical components (Table 1 and Table 2). It has antioxidant properties (Figure 1A), anti-inflammatory properties (Figure 1B), and effects on mitochondrial stability (Figure 1C).

### 4.1. Antioxidant Properties

Oxidative stress occurs when there is an imbalance in the redox reactions, leading to an increase in the production of reactive oxygen species (ROS) [64]. This is harmful to the body, causing inflammation, lipid peroxidation, protein degradation, and damage to other essential molecules [30]. GSE has been found to reduce oxidative stress due to the high concentration of polyphenolic compounds, such as catechin, epicatechin, gallic acid, and proanthocyanidin [25,65,66]. These compounds have the ability to reduce oxidative stress through the direct scavenging of ROS such as hydrogen peroxide, hydroxyl free radicals, and superoxide anions [32,67]. By scavenging reactive oxygen species and reducing nitric oxide levels, GSE prevents oxidative-stress-induced activation of redox-sensitive transcription factors like NF-κB. GSE has been shown to decrease oxidative stress markers including malondialdehyde, an outcome of lipid peroxidation, oxidized LDL, and protein carbonylation while simultaneously increasing total antioxidant capacity and antioxidant enzyme activities including catalase, glutathione peroxidase, superoxide dismutase [43,44,68]. Proanthocyanidins have also been shown to have a more potent free radical scavenging activity compared to vitamins C and E [67].

The proanthocyanidin molecules of GSE scavenge reactive oxygen species, preventing the oxidation of lipids and chelating transition metals such as copper and iron [33]. This effect is prominent in the flavan-3-ols, gallic acids, catechin, and proanthocyanidin compounds of GSE. The antioxidant power of these components can be measured through oxygen radical absorbance capacity, 2,2-diphenyl-1-picrylhydrazyl, and ABTS scavenging assays [69,70,71]. Superoxide is formed initially through aerobic respiration, which can be converted to hydrogen peroxide through enzymatic dismutation. Transition metals like iron are used to reduce hydrogen peroxide, producing highly reactive hydroxyl radicals. These radicals participate in oxidative stress, leading to damage of cellular components such as DNA. The formation of superoxide is associated with conditions such as cancer and neurodegenerative diseases [72,73,74,75]. Inflamed tissues are highly prone to Fenton reactions. Immune cells such as phagocytes release iron from ferritin stores [76]. GSE chelates this iron, preventing the Fenton reaction from occurring [32,77].

Proanthocyanidins are also protective against oxidative damage through the PI3K/Akt signaling pathway, which is a protein cascade involved in cell proliferation, growth, differentiation, protein synthesis, glucose metabolism, cellular migration, and apoptosis [34,78]. GSE proanthocyanidins modulate this protein cascade under different circumstances. During periods of physiologic oxidative stress, GSE proanthocyanidins activate the PI3K/Akt signaling pathway to decrease cellular damage. A study looking into the effect of GSE on neuronal cells showed that pretreatment with GSE promoted the phosphorylation of PI3K/Akt. This reduced ROS generation and inhibited apoptosis [34]. In pathologic causes of oxidative stress, GSE proproanthocyanidins have been shown to decrease PI3K/Akt signaling, preventing cell growth and differentiation. This was shown in a study using pancreatic cancer cells, where GSE prevented progression through the G2 stage of the cell cycle and induced apoptosis [35].

GSE also has the ability to increase gene expression of antioxidant enzymes such as glutathione peroxidase and superoxide dismutase [45]. Studies have shown that a GSE concentration of 12.5 μg/mL was able to decrease mitochondrial damage and significantly reduce intracellular ROS and superoxide levels by 3.5 times [30].

A major consequence of mutant huntingtin (mHTT) expression is a deficiency in peroxisome proliferator-activated receptor gamma coactivator-1⍺ (PGC-1⍺), which is a coregulator for expression of antioxidant enzymes in mitochondria [29]. This leads to increased oxidative stress and damage to DNA as well as striatal degeneration. Current antioxidant management in HD includes the use of free radical scavengers, which consists of enzymatic and non-enzymatic antioxidants. Enzymatic antioxidants include superoxide dismutase, glutathione peroxidase, and catalase, while non-enzymatic antioxidants include ascorbic acid, α-tocopherol, glutathione, retinoic acid, carotenoids, and flavonoids [29]. GSE is associated with both these enzymatic and non-enzymatic antioxidant compounds. Therefore, it is a plausible adjunctive therapy to address oxidative stress in HD.

GSE has been found to prevent the release of proinflammatory mediators including 5-lipoxygenase and prevent an acetylcholine deficit in the brain of patients with Alzheimer disease [46,79,80,81]. GSE also increases nuclear factor erythroid 2-related factor 2 (Nrf2) activity, which is a transcription factor that decreases oxidative stress and the expression of inflammatory cytokines, integrating antioxidant and anti-inflammatory responses [47,82]. Nrf2 is also a major regulator of antioxidant gene expression, acting as a major pathway regulating phase II antioxidant responses when bound to the antioxidant response element after oxidative stress is experienced. Nrf2 has a central role in cell survival, leading to the expression of protective enzymes and scavengers. It was shown to have a key role in Parkinson disease where a deficiency leads to MPTP toxicity with increased microglial activation. Also, Nrf2 overexpression led to an increase in glutathione secretion from astrocytes with models of amyotrophic lateral sclerosis, along with increased survival [83]. GSE has shown to be neuroprotective in neurons due to its interaction with the Nrf2 pathway. GSE primarily interacts with this pathway through two molecular mechanisms. The first is its interaction with Keap1, which contains cysteine residues that when modified result in the stabilization of Nrf2, therefore leading to nuclear accumulation. Once stabilized, this complex combines with Maf, which binds antioxidant response elements located on promoter regions of genes that encode antioxidant and detoxifying enzymes [84]. Here, when GSE is added, it promotes dissociation from Keap1, which allows for nuclear translation and increased antioxidant gene transcription (Figure 1A.). The second mechanism in which GSE interacts with the Nrf2 pathway is its ability to activate AMPK. When AMPK is activated, this phosphorylates p62, which then allows p62 to bind Keap1 [85]. Again, once the Keap1 repressor complex is removed, Nrf2 can enter the nucleus to increase transcription of antioxidant genes [86]. This ultimately creates a positive feedback loop such that actuated NrF2 transcriptionally upregulates p62 expression, which then leads to continuous removal of Keap1 from Nrf2 and so on [85].

This coordinated regulation is relevant in neurodegenerative diseases, which are a state of chronic neuroinflammation [87]. The ability of GSE to suppress inflammatory signaling through inhibition of NF-κB and MAPK pathways, along with increasing Nrf2 activity, suggests a potential role in attenuating oxidation-driven neurodegeneration in HD [36].

### 4.2. Anti-Inflammatory Properties

GSE has both direct and indirect anti-inflammatory properties. There are three primary direct mechanisms where GSE has shown anti-inflammatory properties. The first is its ability to directly block NF-κB activation. GSE inhibits the phosphorylation of IκB kinase (IKKα/β), preventing degradation of IκBα and blocking nuclear translocation of the NF-κB p65 subunit. This prevents the transcription of proinflammatory cytokines and enzymes, including TNF-α, IL-1β, IL-6, IL-8, COX-2, and iNOS (Figure 1B), decreasing inflammation [37,38,39,40]. The second method is through the ability of GSE to inhibit the MAPK pathway. The MAP kinases ERK1/2, JNK, and p38 are critical to the amplification of the stress response [88]. In regard to UVB-induced photocarcinogenesis, GSE was found to reactivate MAP kinase phosphatase (MKP), which dephosphorylates MAP kinases as a method of negative regulation [49]. Finally, GSE has been found to be involved with STAT protein modulation. GSE directly inhibits the phosphorylation of both STAT3 (Tyr705) and STAT3 (Ser727), which suppresses Th17 cell differentiation and reduces proinflammatory responses. GSE was also shown to increase STAT5 activity, leading to more regulatory T-cells and an anti-inflammatory response [50].

Indirect mechanisms of GSE tend to follow the gut–brain axis. First, GSE has the ability to alter the gut microbiome. GSE has been shown to alleviate dextran-sulfate-sodium-induced colitis through decreasing proinflammatory bacteria, including Bacteroidetes, Dubosiella, Veillonella, and increasing more beneficial organisms such as Akkermansia [48]. Akkermansia has the ability to degrade mucin into short-chain fatty acids, which are a part of the regulation of microglial homeostasis and function [48,89]. The colonic microbiome can also utilize the proanthocyanidins in GSE that are not directly absorbed to create more bioavailable metabolites, such as 5-(3′,4′-dihydroxyphenyl)-γ-valerolactone. This can activate the Nrf2 pathway which engages cellular defenses and decreases stress responses, reducing inflammatory responses [90]. Decreasing systemic inflammation also minimizes the potential triggers for neuroinflammation, as gastrointestinal inflammation has been shown to be related to neurological disorders [91].

Before HD is diagnosed clinically, mHTT activates microglial cells and astrocytes, creating a state of neuroinflammation [87]. The activation of astrocytes and microglial cells leads to the release of proinflammatory cytokines such as IL-6, IL-8, IL-4, IL-10, and TNF-α. Chronically, this increases neuronal vulnerability and accelerates disease progression [92]. Specifically for neuroinflammation present in HD, GSE has direct anti-neuroinflammatory effects. First, GSE and its metabolites have been shown to cross the blood–brain barrier [93]. Once there, GSE has the ability to reduce microglia from the proinflammatory M1 phenotype to the anti-inflammatory M2 phenotype. This is done through the modulation of the PI3K/AKT pathway, where TLR4 and NF-κB expression is altered [51]. GSE is also able to act within the microglia and astroglia to block protein overexpression and is able to restore sirtuin levels [94]. This is relevant for HD because sirtuin is a neuroprotective protein that has been studied as an additional potential treatment for HD [95]. GSE was also found to reduce proinflammatory markers such as JNK, IL-6, and TNF-α expression in the hippocampus [52]. Specific metabolites, such as anthocyanins and cyanidin glycoside, of GSE cross the blood–brain barrier [93]. These smaller molecules accumulate in the brain of Sprague Dawley rats. However, the heterogeneous GSE polyphenolics have different abilities to cross the blood–brain barrier. Catechin and epicatechin were detected in brain tissue of Sprague Dawley rats while larger polymeric proanthocyanidins were not detected in substantial concentrations [96]. Current evidence from animal studies suggest that GSE polyphenolics cross the blood–brain barrier, but extrapolation to humans and individual polyphenolic bioavailability in the brain requires further investigation. Therefore, the therapeutic concentration for effect in HD patients and the oral dose required to reach this concentration remain a limitation of studying GSE in HD patients.

### 4.3. Mitochondrial Support

GSE has been shown to provide support to mitochondrial structure and function. One way GSE provides mitochondrial support is by blocking mitochondria-associated oxidative stress and therefore restoring resting membrane potential. This occurs by blocking the mitochondrial permeability transition pore (mPTP) from opening, therefore preventing depolarization and apoptosis. The opening of the mPTP causes mitochondrial swelling, release of proapoptotic proteins, and uncoupling of mitochondrial oxidative phosphorylation [97]. This pore regulates the transport of calcium ions and mitochondrial reactive oxygen species [98]. GSE has been shown to act on the mPTP mechanism to preserve mitochondrial integrity and slow neuronal loss by increasing the mPTP threshold for opening [97]. At the molecular level, this process involves increased phosphorylation of GSK-3β, which then binds to the adenine nucleotide translocator (ANT). This interaction then prevents the formation of the ANT–cyclophilin D complex, helping maintain mitochondrial membrane potential and ATP production (Figure 1C). By stabilizing mitochondrial function, GSE may reduce neuronal susceptibility to energy failure and apoptosis [97].

Similar protective effects have been observed in mouse neuroblastoma N2a cells subjected to reoxygenation injury, where GSE restored mitochondrial membrane potential and increased ATP generation [99]. These findings were accomplished through the ability of GSE to act as an antioxidant and also reduce mitochondrial endoplasmic reticulum stress by downregulating the transcription of CHOP, GRP78, and caspase-12, which all are involved in the apoptosis stress response [99]. By downregulating proapoptotic transcription factors, GSE supports the recovery of mitochondrial membrane potential and reduces apoptosis. However, GSE has the opposite effect in cancer cells, where it induces apoptosis by inhibiting mitochondrial electron transport chain complex III activity and glutathione activity [41].

GSE also impacts mitochondrial calcium influx and release, which has a direct effect on mitochondrial ATP production through interactions with molecules in the Krebs cycle [100]. In hippocampal mitochondria, GSE indirectly regulates calcium influx and release, protecting against calcium-mediated mitochondrial damage including the formation of the permeable transition pore [97]. In excited hippocampal neurons, GSE has been shown to prevent the calcium influx that normally occurs with glutamate excitation, suggesting a role in calcium transport regulation [42]. However, GSE again demonstrates a different effect in cancer cells, increasing mitochondrial calcium influx [31].

### 4.4. Effects on Cognition

GSE has also been shown to have a positive effect on neurogenesis and cognition [101]. Neurogenesis in the adult brain takes place in the subventricular and subgranular zones, and a higher rate of neurogenesis corresponds to higher cognition. Neurogenesis results through a brain-derived neurotrophic factor (BDNF) and GSE has been shown to increase BDNF through the neurotrophin pathway, thus increasing neurogenesis. GSE is said to have an “anti-aging” effect because of its ability to increase BDNF, which then increases the synaptophysin protein. This protein supports synaptogenesis and therefore long-term potentiation, making it essential for synapse formation and cognitive function [53]. These findings suggest that GSE may not only mitigate neurodegenerative processes but also support compensatory neural plasticity.

Collectively, the antioxidant, anti-inflammatory, mitochondrial-stabilizing, and neurotrophic properties of GSE make it a biologically plausible multi-target intervention for neurodegenerative diseases. These mechanisms suggest that GSE interacts with multiple pathological pathways implicated in HD, supporting its consideration as a disease-modifying adjunct rather than solely a symptomatic intervention.

## 5. Evidence of Efficacy of Treatment

While GSE remains relatively understudied in HD, emerging preclinical evidence suggests it may reduce mHTT/polyglutamine (polyQ) aggregation and improve disease phenotypes. Wang et al. first demonstrated this effect using a *Drosophila* model, reporting that GSE significantly inhibited polyQ aggregation in an inducible pheochromocytoma cell model expressing a huntingtin fragment fused to EGFP [13]. In the same line of investigation, treatment with GSE extended the lifespan of elav>Q93htt exon1 flies [13]. Similar findings were reported in a separate *Drosophila* study, where GSE supplementation improved lifespan and locomotor function and reduced neurodegeneration [102]. Importantly, Wang et al. also translated these findings into a mammalian system by testing GSE in a mouse model of HD. In this experiment, transgenic mice received 100 mg/kg/day of GSE, resulting in attenuated motor skill decline and extended lifespan compared with placebo [13]. Because R6/2 mice develop a robust and rapidly progressive HD phenotype, the observation of both motor and survival benefits strengthens the interpretation that GSE may influence underlying disease mechanisms rather than simply producing non-specific behavioral effects. Collectively, these studies demonstrate that GSE exhibits neuroprotective effects across multiple experimental models relevant to HD.

These findings align with the broader HD literature, which increasingly highlights oxidative stress and impaired protein homeostasis as key therapeutic targets. Supporting this concept, a review of antioxidants in HD describes GSPE-mediated inhibition of mHTT aggregation in PC-12 cells expressing Htt103Q-EGFP, along with reduced oxidative damage induced by mHTT expression [29]. These results suggest that GSPE may exert dual actions by limiting aggregation while simultaneously reducing oxidative injury. In addition, research on polyphenols in neurodegenerative disease provides further mechanistic plausibility. Polyphenolic compounds have been shown to influence pathways involved in proteinopathies, including autophagy and proteostasis, mitochondrial dysfunction, and neuroinflammation—processes that are strongly implicated in HD pathogenesis [103,104,105,106]. Taken together, the available preclinical evidence indicates that GSPE/GSE can (1) reduce aggregation and oxidative damage in cellular HD models, (2) improve survival in HD fly models, and (3) improve motor decline and survival in HD mouse models.

Although clinical trials specifically evaluating GSE in HD have not yet been conducted, human studies of grape-derived polyphenols provide important translational context. Several randomized controlled trials have demonstrated that GSE supplementation can influence oxidative stress and inflammatory biomarkers, mechanisms that overlap with established HD pathophysiology. For example, an 8-week supplementation study reported improvements in glutathione and malondialdehyde levels, suggesting beneficial effects on redox balance and lipid peroxidation in humans [54]. Additional trials have similarly reported reductions in markers associated with oxidative stress, though inflammatory outcomes appear more heterogeneous across populations and study designs [54,55,56,57,58]. Systematic reviews of randomized trials involving grape polyphenols also report measurable changes in inflammatory mediators such as CRP, IL-6, and TNF-α, although effect sizes vary depending on product formulation, dosage, and baseline inflammatory status [90,107,108,109]. Beyond biomarker outcomes, human trials have reported physiologic effects consistent with improved endothelial function and vascular tone, including enhanced flow-mediated dilation and modest reductions in blood pressure [59]. While these findings do not establish efficacy in HD specifically, they demonstrate that orally administered GSE can engage relevant biological pathways in humans and therefore support the rationale for HD-focused clinical trials incorporating oxidative stress, inflammatory, and functional endpoints.

## 6. Safety and Side Effects

Mild side effects have been associated with GSE, providing a favorable safety profile. Common short-term side effects documented in a variety of clinical trials have shown itching, dizziness, sore throat, and cough [110]. Furthermore, no serious adverse events were consistently reported in clinical trials involving antioxidant and anti-inflammatory properties of GSE involved in glycemic control [54,58,111,112,113,114,115]. A trial was done on healthy participants using 1000–2500 mg of GSE orally everyday for four weeks to monitor for adverse effects. No participant was found to have significant adverse effects or abnormal vital signs. The mild effects of constipation, nausea, diarrhea, and headache were found to be transient and unlikely to be associated with GSE consumption [116]. Research using GSE has shown a typical dosing ranging from 300–600 mg/day for 4–12 weeks, and there have been no adverse events leading to study discontinuation [57]. At high doses, GSE was found to have no adverse effects on food intake, organ weights, or hematology except a slight increase in platelets [43]. However, the GSE safety profile is not sufficiently known due to the lack of adequate long-term data [110].

Furthermore, while short-term side effects are mild, there needs to be more research regarding the long-term consequences of GSE supplementation as this will likely be a life-long medication for HD patients. Current research has yet to yield significant evidence around the efficacy or safety of GSE in HD-positive pregnant women and whether there are teratogenic effects toward the fetus. However, the biological effects of polyphenols in general populations of pregnant women have started to be investigated. Research indicated a favorable association with polyphenols regarding the health of pregnant women, reducing inflammatory burden and metabolic regulation [4]. Outcomes such as these may provide insight to similar disease pathologies, such as HD. Flavonoid polyphenols have also demonstrated the ability to cross biological barriers, such as the placenta [117]. This ability raises more interest in the functional possibilities of the neuroprotective effects it may possess and also raises concerns for the health of the fetus [117]. Two important factors to consider include dosage and timing of exposure. In some reports, excessive polyphenol intake in late pregnancy has resulted in adverse cardiovascular fetal health, including constriction of the ductus arteriosus [118].

GSE is a concentrated source of flavonoid polyphenol components that have been shown to modulate the inflammatory process of gestational diabetes mellitus. Findings such as these provide support for exploration of GSE as a potential modulator of oxidative and inflammatory pathways in HD. Though pregnancy-based studies cannot be directly generalized to neurodegenerative disease, they provide a valuable understanding of the beneficial activity of polyphenols. Collectively, mechanistic actions of polyphenols established previously indicate an overlap in benefits to many different inflammatory diseases [4].

While GSE potentially could be beneficial in treating HD, it is important to consider potential drug–drug interactions with current HD therapies. Current HD therapy is designed for symptom management, as there is no curative treatment. Because of this, there is variability in the standard of care for HD treatment. The most frequently used agents for HD symptom management utilize various CYP pathways, including CYP2D6, CYP3A4, CYP1A2, and CYP2C19. For example, tetrabenazine and deutetrabenazine are metabolized by CYP2D6; if GSE affects this pathway, drug exposure may be altered [119]. Investigating the mechanisms through which GSE may influence these pathways is an important step in determining its safety. One experimental study demonstrated that grape seed inhibits CYP2C9 and CYP3A4 at 10 μM [119], raising a potential concern for altered drug metabolism regarding the HD pharmacological medications that utilize these pathways. Another study completed on the effects of GSE, specifically CYP3A4, evaluated nine different commercial GSE products [120]. The study found that four brands of GSE showed no effect, whereas five showed variable inhibition of CYP3A4, ranging from 6.4% to 26.8%. Due to the heterogeneity introduced by GSE preparation and development, this data provides insight into how different extraction methods and phytochemical composition may lead to varying effects on the pathway observed in this study [120].

However, the current evidence for clinically meaningful GSE drug interactions remains limited due to a lack of clinical studies. Therefore, further investigation is needed to define the impact of GSE on drug-metabolizing enzymes, particularly in polypharmacy settings relevant to HD management.

## 7. Future Directions and Challenges

While GSE has shown to be promising as an adjunctive treatment for HD, several challenges must be addressed before transitioning into clinical practice. The most significant challenge is the absence of HD-specific clinical trials. Future research should prioritize early-phase studies to assess safety, tolerability, and biological target engagement in HD patients. Research should also target those with genetically confirmed HD patients who are asymptomatic in order to prevent disease progression. Standardization also remains a major challenge as GSE formulations (Table 2) vary widely in polyphenol composition based on grape source and different extraction methods. Future trials should be directed to determine a quantified therapeutic concentration that can be consistent across extraction methods. Finally, while short-term safety data for GSE is favorable, long-term use in HD populations remains unstudied. Potential interactions and cumulative effects warrant careful monitoring. While challenges related to formulation variability, bioavailability, and long-term safety remain, the convergence of mechanistic rationale and preclinical efficacy supports targeted clinical evaluation. Ultimately, GSE is best considered as a low-risk adjunct that may complement emerging disease-modifying therapies rather than replace them.

## 8. Conclusions

HD remains a progressive neurodegenerative disorder for which current treatments provide only symptomatic benefit. Growing evidence implicates oxidative stress, neuroinflammation, mitochondrial dysfunction, and impaired protein homeostasis as key drivers of disease progression. GSE, a polyphenol-rich compound, directly targets these pathological mechanisms through antioxidant, anti-inflammatory, mitochondrial-stabilizing, and neurotrophic effects. Preclinical studies across cellular, Drosophila, and murine HD models demonstrate that GSE reduces mutant HTT aggregation, improves motor performance, and extends survival. Although HD-specific clinical trials are lacking, human studies of GSE and grape-derived polyphenols show modulation of oxidative stress and inflammatory pathways relevant to HD pathogenesis. Importantly, GSE exhibits a favorable short-term safety profile, supporting further investigation. In summary, GSE represents a promising multi-target adjunctive strategy that aligns with current understanding of HD biology. Continued rigorous investigation may clarify its potential role in slowing disease progression and improving patient outcomes.

## Figures and Tables

**Table 1:** Summary of GSE Effects Related to HD in Various Model Systems.

**Cells and Cell Line Models**			
**Grape Seed Extract**	**Model**	**Outcome(s)**	**References**
Changsha Huir Biological-Tech Crp. Ltd. GSE (Changsha, China) In-house derived GSE	Caco-2 human colon cell line	↓ oxidative stress and intestinal inflammation Causes apoptosis in cancer cells	[30] [31]
InterHealth Nutraceuticals Inc. (Benicia, CA, USA)	Embryonic chick cardiomyocytes	Contributes to cardioprotection against oxidant injury	[32]
In-house derived CPE and procyanidin fractions (Campbell Early)	HepG2 human hepatocellular carcinoma cell line	Protects against oxidative damage	[33]
Solarbio (Beijing, China)	PC12 rat pheochromocytoma cell line	↓ ROS and inhibits apoptosis (↓ oxidative damage)	[34]
Kikkoman Corporation (Noda, Japan)	AsPC-1, PANC-1, and Miapaca-2 human pancreatic cell lines	↓ cell proliferation, ↑ tumor cell apoptosis → possibly chemotherapeutic in pancreatic cancer	[35]
In-house derived GSE Tianjin Institute JF-NATURE (Tianjin, China) Les Derives Resiniques et Terpeniques (Dax, France) Grape seed PAC powder Shandong Shengjiade Biotechnology Co., Ltd. (Shandong, China)	RAW264.7 murine leukemia macrophages	↓ inflammatory cytokines Anti-inflammatory and immunomodulatory Modulates inflammatory response ↓ inflammatory response through inhibition of NF-κB	[36] [37] [38] [39]
Hanlim Pharmaceutical (Seoul, Korea)	Hepatic stellate cells derived from LX-2 hematopoietic stem cell line	↓ inflammatory response through inhibition of NF-κB	[40]
ActiVin San Joaquin Valley Concentrates (Fresno, CA, USA)	FaDu human squamous cell carcinoma cell line and Detroit 562 human pharyngeal cancer cell line	↑ oxidative/metabolic stress in cancer cells → apoptosis and autophagy	[41]
IH636 InterHealth Nutraceuticals (Benicia, USA)	Sprague Dawley rat hippocampal neuron culture	Protects against oxygen–glucose-deprivation-induced neurotoxicity	[42]
**Animal Models**			
**Grape Seed Extract**	**Model**	**Outcome(s)**	**References**
Inhouse derived from Carignan Northern Tunisia	Healthy Wistar rats	Antioxidant and anti-inflammatory properties, ↑ neurogenesis	[43]
Les Derives Resiniques et Terpeniques (Dax, France)	LPS-exposed Wistar rats	Antioxidant and anti-inflammatory properties	[44]
Grape seed powder *Vitis labruscana* Bailey Campbell Early	Sprague Dawley Rats	↓ lipid peroxidases, ↑ ratio of reduced glutathione and oxidized glutathione	[45]
Natural Sourcing, LLC (Oxford, USA) cold press method	Wistar rat model of Alzheimer	↑ spatial memory performance and acetylcholine levels	[46]
Zelang Medical Technology Company (Nanjing, China)	Aflatoxin-B1-exposed Cobb broilers	↑ antioxidant defense system through Nrf2 and ↓ inflammatory cytokines → alleviates AFB1-induced immunotoxicity	[47]
Grape seed PAC powder Shandong Shengjiade Biotechnology Co., Ltd. (Shandong, China) Grape seed extract (Shanghai Yuanye Bio-Technology Co., Ltd., Shanghai China)	C57BL/6 mouse dextran-sodium-sulfate-induced colitis	↓ inflammatory response through inhibition of NF-κB ↓ inflammatory cytokines and oxidative stress, maintains intestinal barrier, and balances microbial community	[39] [48]
Kikkoman Corporation (Noda, Japan)	UVB-exposed SKH-1 hairless mice	Protects skin from effects of UVB radiation through MAPK and NF-κB	[49]
Hanlim Pharmaceutical company (Seoul, Korea)	C57BL/6 mouse model of obesity-associated collagen-induced arthritis	↑ regulatory T cells and ↓ Th17 cells through STAT protein regulation	[50]
Solarbio (Beijing, China)	Sprague Dawley rat spinal cord injury	Regulates microglial polarization and prevents neuronal apoptosis	[51]
Les Derives Resiniques et Terpeniques (Dax, France)	Wistar rat cafeteria diet	↓ inflammatory activation, conserves mitochondrial function through ↑ antioxidants	[52]
Adonis gol darou company (Tehran, Iran)	Aged BALB/c mice	Improves memory and learning performance, ↑ neurogenesis/synaptogenesis	[53]
**Human Studies**			
**Grape Seed Extract**	**Model**	**Outcome(s)**	**References**
Barij Essence Pharmaceutical, (Tehran, Iran)	Female athletes	↑ glutathione and insulin sensitivity, ↓ malondialdehyde and insulin	[54]
Drug Applied Research Center (Tabriz, Iran).	Patients with mild to moderate hyperlipidemia	↑ apo-AI, HDL-C, and paraoxonase, ↓ total cholesterol, triglycerides, and LDL-C → possible role in prevention of atherosclerosis	[55]
OMNIVIN-20R, Ajinomoto-Omnichem (Wettern, Belgium)	Adult patients with obesity	↑ antioxidant activity	[56]
MegaNatural Gold Extract; Polyphenolics (Fresno, USA)	Patients with type 2 diabetes	↓ total cholesterol, fructosamine, C-reactive protein → ↓ inflammatory/glycemic markers	[57]
Carignan (Northern Tunisia)	Patients with chronic kidney disease	↓ inflammation and proteinuria, ↑ GFR and antioxidant activity, and counteracted anemia/thrombocytopenia	[58]
Kikkoman Corporation (Noda, Japan)	Patients with prehypertension	↓ systolic BP, diastolic BP, stiffness, elastic modulus, and pulse wave velocity in non-smokers	[59]

**Table 2:** Heterogeneous Chemical Composition of GSE Used in Referenced Studies.

	Extraction Solvent	Reported Analytical Results	Reference(s)
*Vitis vinifera*, Merlot *Vitis vinifera*, Carignan *Vitis vinifera*, Syrah	80% ethanol, 20% water	Syrah GSE; 4.14% total phenolic acids, 11.58% total flavonol, 73.58% total flavan-3-ols Merlot GSE; 8.67% total phenolic acid, 10.82% total flavonol, 60.84% total flavan-3-ols Carignan GSE; 7.16% total phenolic acid, 16.37% total flavonol, 57.86% total flavan-3-ols Further characterization was available	[36]
Italia white grape Palieri red grape Red Globe red grape (Puglia region, Italy)	70% methanol, 29% water, 1% formic acid	Italia; 2.5 mg/g catechins, 4.1 mg/g procyanidins Palieri; 6.2 mg/g catechins, 5.6 mg/g procyanidins Red Globe; 3.9 mg/g catechins, 3.9 mg/g procyanidins	[31]
IH636 grape seed proanthocyanidin extract (ActiVin, InterHealth Nutraceuticals, Inc., Benicia, USA)	Unclear	75–80% oligomeric proanthocyanidins and 3–5% monomeric proanthocyanidins	[32,41,42]
Vitis vinifera, Campbell Early	75% acetone, 25% water followed by 80% methanol, 20% water	Crude polyphenolic extract; 65.0% polyphenols, 9.5% flavan-3-ols, 19.3% polymeric procyanidins Oligomeric procyanidin fraction; 60.9% polyphenols, 24.8% flavan-3-ols, 8.4% polymeric procyanidins Polymeric procyanidin fraction; 84.7% polyphenols, 9.5% flavan-3-ols, 37.2% polymeric procyanidins	[33]
Solarbio (Beijing, China)	Unclear	>95% pure	[34,51]
OMNIVIN-20R (Ajinomoto Omnichem, Wettern, Belgium)	Unclear	10.9 mmol/g total phenol	[56,60]
Changsha Huir Biological-Tech Crp. Ltd. (Changsha, China)	25% ethanol, 75% water	36% total polyphenolics (dry mass) 28% proanthocyanidins LC-MS showed distribution but not quantity of proanthocyanidins and gallates	[30]
Carignan (Northern Tunisia)	10% ethanol, 90% water	Total phenolics 67 mg/g extract, total flavanoids 16 mg/g extract, further breakdown provided in Charradi et al., 2012. [61]	[43,61]
Carignan (Northern Tunisia)	10% ethanol, 90% water	Main components were 2,5-dihydroxybenzoic acid (41.26%), gallic acid (41.53%), and vanillin (9.21%), other components were less than 2%	[58,62]
Kikkoman Corp (Noda, Japan), also known as Gravinol^TM^	Unclear	89% total proanthocyanidins present mainly as oligomers (74.8%), but also dimers, trimers, and tetramers. 6.6% flavanols	[35,49,59]
MegaNatural Gold Extract (Polyphenolics, Fresno, USA)	Unclear	92.0% (w/w) total phenolics; 19.8% monomers, 69.8% oligomers, 10.3% polymers	[57,63]
Hanlim Pharmaceuticals (Seoul, Korea)	Unclear	80% proanthocyanidins; several catechin monomers	[40,50]
Drug Applied Research Center (Tabriz, Iran)	Unclear	95% proanthocyanidins 80% other polyphenolics	[55]
Barij Essence Pharmaceutical (Tehran, Iran)	Unclear	Not quantitated, but “major components” were flavonoids, linoleic acid, phenolic procyanidins, and vitamins C and E	[54]
Adonis gol darou company (Tehran, Iran)	Unclear	>95% proanthocyanidins, no other information available	[53]
Vitaflavan (Les Derives Resiniques et Terpeniques, Dax, France)	Unclear	Monomeric (16.6–21.3%), dimeric (17.4–18.8%), trimeric (16.0–16.3%), tetrameric (9.3–13.3%) and oligomeric (31.7–35.7%) proanthocyanidins, and phenolic acids (4.2–4.7%) 78.4% proanthocyanidins containing 41.6% dimers and trimers, 21.6% flavan-3-ol monomers	[38,44] [52]
Tianjin Institute of JF-NATURE (Tianjin, China)	Unclear	95% pure containing 90% polyphenols; 85% oligomeric proanthocyanidins and >7% catechin and epicatechin	[37]
Grape seed PAC powder (Shandong Shengjade Bio-Technology Co., Ltd., Shandong, China)	Unclear	>98% pure, no other data available	[39]
Shanghai Yuany Bio-Technology Co., Ltd. (Shanghai, China)	Unclear	>98% pure mainly oligomeric proanthocyanidins, no other data available	[48]
Grape seed extract (Zelang Medical Technology Co., Nanjing, China)	Unclear	>98% pure, no other data available	[47]
Grape seed oil (Natural Sourcing, LLC, Oxford, USA)	Not available	Not available	[46]
Grape seed powder, *Vitis labruscana* Bailey, Campbell Early (Gyeongsan-si, Gyeongsangbuk-do, Korea)	Not available	Not available	[45]

## Data Availability

No new data were created or analyzed in this study. Data sharing is not applicable to this article.

## References

[1] Ajitkumar A., Lui F., De Jesus O. (2026). Huntington Disease. StatPearls.

[2] Reiner A., Dragatsis I., Dietrich P. (2011). Genetics and Neuropathology of Huntington’s Disease. International Review of Neurobiology.

[3] Ross C.A., Aylward E.H., Wild E.J., Langbehn D.R., Long J.D., Warner J.H., Scahill R.I., Leavitt B.R., Stout J.C., Paulsen J.S. (2014). Huntington Disease: Natural History, Biomarkers and Prospects for Therapeutics. Nat. Rev. Neurol..

[4] Jimenez-Sanchez M., Licitra F., Underwood B.R., Rubinsztein D.C. (2017). Huntington’s Disease: Mechanisms of Pathogenesis and Therapeutic Strategies. Cold Spring Harb. Perspect. Med..

[5] Dorsey E.R., Beck C.A., Darwin K., Nichols P., Brocht A.F.D., Biglan K.M., Shoulson I. (2013). Huntington Study Group COHORT Investigators Natural History of Huntington Disease. JAMA Neurol..

[6] Rosenblatt A., Liang K.-Y., Zhou H., Abbott M.H., Gourley L.M., Margolis R.L., Brandt J., Ross C.A. (2006). The Association of CAG Repeat Length with Clinical Progression in Huntington Disease. Neurology.

[7] Purdon S.E., Mohr E., Ilivitsky V., Jones B.D. (1994). Huntington’s Disease: Pathogenesis, Diagnosis and Treatment. J. Psychiatry Neurosci..

[8] Estarellas M., Gomis C., Canals J.M. (2026). The Evolving Landscape of Stem Cell Therapies for Huntington’s Disease. Mol. Diagn. Ther..

[9] Farag M., Tabrizi S.J., Wild E.J. (2025). Huntington’s Disease Clinical Trials Update: March 2025. J. Huntington’s Dis..

[10] Yang W. (2002). Aggregated Polyglutamine Peptides Delivered to Nuclei Are Toxic to Mammalian Cells. Hum. Mol. Genet..

[11] Ross C.A., Tabrizi S.J. (2011). Huntington’s Disease: From Molecular Pathogenesis to Clinical Treatment. Lancet Neurol..

[12] Bates G.P., Dorsey R., Gusella J.F., Hayden M.R., Kay C., Leavitt B.R., Nance M., Ross C.A., Scahill R.I., Wetzel R. (2015). Huntington Disease. Nat. Rev. Dis. Primers.

[13] Wang J., Pfleger C., Friedman L., Vittorino R., Zhao W., Qian X., Conley L., Ho L., Pasinetti G. (2010). Potential Application of Grape Derived Polyphenols in Huntington’s Disease. Transl. Neurosci..

[14] Gupta M., Dey S., Marbaniang D., Pal P., Ray S., Mazumder B. (2020). Grape Seed Extract: Having a Potential Health Benefits. J. Food Sci. Technol..

[15] Matthäus B. (2008). Virgin Grape Seed Oil: Is It Really a Nutritional Highlight?. Eur. J. Lipid Sci. Technol..

[16] Duba K.S., Fiori L. (2015). Supercritical CO2 Extraction of Grape Seed Oil: Effect of Process Parameters on the Extraction Kinetics. J. Supercrit. Fluids.

[17] Hegedüs I., Andreidesz K., Szentpéteri J.L., Kaleta Z., Szabó L., Szigeti K., Gulyás B., Padmanabhan P., Budan F., Máthé D. (2022). The Utilization of Physiologically Active Molecular Components of Grape Seeds and Grape Marc. Int. J. Mol. Sci..

[18] Król A., Amarowicz R., Weidner S. (2014). Changes in the Composition of Phenolic Compounds and Antioxidant Properties of Grapevine Roots and Leaves (*Vitis vinifera* L.) under Continuous of Long-Term Drought Stress. Acta Physiol. Plant..

[19] Chacón-Vozmediano J.L., Martínez-Gascueña J., García-Romero E., Gómez-Alonso S., García-Navarro F.J., Jiménez-Ballesta R. (2021). Effects of Water Stress on the Phenolic Compounds of ‘Merlot’ Grapes in a Semi-Arid Mediterranean Climate. Horticulturae.

[20] Teixeira A., Eiras-Dias J., Castellarin S., Gerós H. (2013). Berry Phenolics of Grapevine under Challenging Environments. Int. J. Mol. Sci..

[21] Yamakoshi J., Saito M., Kataoka S., Kikuchi M. (2002). Safety Evaluation of Proanthocyanidin-Rich Extract from Grape Seeds. Food Chem. Toxicol..

[22] Stratakos A., Koidis A., Preedy V. (2015). Methods for Extracting Essential Oils. Essential Oils in Food Preservation, Flavour and Safety.

[23] Shi J., Yu J., Pohorly J.E., Kakuda Y. (2003). Polyphenolics in Grape Seeds—Biochemistry and Functionality. J. Med. Food.

[24] Gómez-Plaza E., Gil-Muñoz R., López-Roca J.M., Martínez-Cutillas A., Fernández-Fernández J.I. (2001). Phenolic Compounds and Color Stability of Red Wines: Effect of Skin Maceration Time. Am. J. Enol. Vitic..

[25] Yilmaz Y., Toledo R.T. (2004). Major Flavonoids in Grape Seeds and Skins: Antioxidant Capacity of Catechin, Epicatechin, and Gallic Acid. J. Agric. Food Chem..

[26] Alkhedaide A., Alshehri Z.S., Sabry A., Abdel-Ghaffar T., Soliman M.M., Attia H. (2016). Protective Effect of Grape Seed Extract against Cadmium-Induced Testicular Dysfunction. Mol. Med. Rep..

[27] Rombaut N., Savoire R., Thomasset B., Hecke E., Lanoisellé J.-L. (2015). Optimization of Oil Yield and Oil Total Phenolic Content during Grape Seed Cold Screw Pressing. Ind. Crops Prod..

[28] Garavaglia J., Markoski M.M., Oliveira A., Marcadenti A. (2016). Grape Seed Oil Compounds: Biological and Chemical Actions for Health. Nutr. Metab. Insights.

[29] Johri A., Beal M.F. (2012). Antioxidants in Huntington’s Disease. Biochim. Biophys. Acta (BBA)-Mol. Basis Dis..

[30] Nallathambi R., Poulev A., Zuk J.B., Raskin I. (2020). Proanthocyanidin-Rich Grape Seed Extract Reduces Inflammation and Oxidative Stress and Restores Tight Junction Barrier Function in Caco-2 Colon Cells. Nutrients.

[31] Dinicola S., Mariggiò M.A., Morabito C., Guarnieri S., Cucina A., Pasqualato A., D’Anselmi F., Proietti S., Coluccia P., Bizzarri M. (2013). Grape Seed Extract Triggers Apoptosis in Caco-2 Human Colon Cancer Cells through Reactive Oxygen Species and Calcium Increase: Extracellular Signal-Regulated Kinase Involvement. Br. J. Nutr..

[32] Shao Z., Becker L., Vanden Hoek T., Schumacker P., Li C.-Q., Zhao D., Wojcik K., Anderson T., Qin Y., Dey L. (2003). Grape Seed Proanthocyanidin Extract Attenuates Oxidant Injury in Cardiomyocytes. Pharmacol. Res..

[33] Kim Y., Choi Y., Ham H., Jeong H.-S., Lee J. (2013). Protective Effects of Oligomeric and Polymeric Procyanidin Fractions from Defatted Grape Seeds on Tert-Butyl Hydroperoxide-Induced Oxidative Damage in HepG2 Cells. Food Chem..

[34] He X., Guo X., Ma Z., Li Y., Kang J., Zhang G., Gao Y., Liu M., Chen H., Kang X. (2021). Grape Seed Proanthocyanidins Protect PC12 Cells from Hydrogen Peroxide-Induced Damage via the PI3K/AKT Signaling Pathway. Neurosci. Lett..

[35] Prasad R., Vaid M., Katiyar S.K. (2012). Grape Proanthocyanidin Inhibit Pancreatic Cancer Cell Growth In Vitro and In Vivo through Induction of Apoptosis and by Targeting the PI3K/Akt Pathway. PLoS ONE.

[36] Harbeoui H., Hichami A., Wannes W.A., Lemput J., Tounsi M.S., Khan N.A. (2019). Anti-Inflammatory Effect of Grape (*Vitis vinifera* L.) Seed Extract through the Downregulation of NF-κB and MAPK Pathways in LPS-Induced RAW264.7 Macrophages. S. Afr. J. Bot..

[37] Chu H., Tang Q., Huang H., Hao W., Wei X. (2016). Grape-Seed Proanthocyanidins Inhibit the Lipopolysaccharide-Induced Inflammatory Mediator Expression in RAW264.7 Macrophages by Suppressing MAPK and NF-Κb Signal Pathways. Environ. Toxicol. Pharmacol..

[38] Terra X., Valls J., Vitrac X., Mérrillon J.-M., Arola L., Ardèvol A., Bladé C., Fernández-Larrea J., Pujadas G., Salvadó J. (2007). Grape-Seed Procyanidins Act as Antiinflammatory Agents in Endotoxin-Stimulated RAW 264.7 Macrophages by Inhibiting NFkB Signaling Pathway. J. Agric. Food Chem..

[39] Chu L., Zhang S., Wu W., Gong Y., Chen Z., Wen Y., Wang Y., Wang L. (2024). Grape Seed Proanthocyanidin Extract Alleviates Inflammation in Experimental Colitis Mice by Inhibiting NF-κB Signaling Pathway. Environ. Toxicol..

[40] Lee J.-W., Kim Y.I., Kim Y., Choi M., Min S., Joo Y.H., Yim S.-V., Chung N. (2017). Grape Seed Proanthocyanidin Inhibits Inflammatory Responses in Hepatic Stellate Cells by Modulating the MAPK, Akt and NF-κB Signaling Pathways. Int. J. Mol. Med..

[41] Shrotriya S., Deep G., Lopert P., Patel M., Agarwal R., Agarwal C. (2015). Grape Seed Extract Targets Mitochondrial Electron Transport Chain Complex III and Induces Oxidative and Metabolic Stress Leading to Cytoprotective Autophagy and Apoptotic Death in Human Head and Neck Cancer Cells. Mol. Carcinog..

[42] Ahn S.-H., Kim H.J., Jeong I., Hong Y.J., Kim M.-J., Rhie D.-J., Jo Y.-H., Hahn S.J., Yoon S.H. (2011). Grape Seed Proanthocyanidin Extract Inhibits Glutamate-Induced Cell Death through Inhibition of Calcium Signals and Nitric Oxide Formation in Cultured Rat Hippocampal Neurons. BMC Neurosci..

[43] Charradi K., Mahmoudi M., Bedhiafi T., Jebari K., El May M.V., Limam F., Aouani E. (2018). Safety Evaluation, Anti-Oxidative and Anti-Inflammatory Effects of Subchronically Dietary Supplemented High Dosing Grape Seed Powder (GSP) to Healthy Rat. Biomed. Pharmacother..

[44] Pallarès V., Fernández-Iglesias A., Cedó L., Castell-Auví A., Pinent M., Ardévol A., Salvadó M.J., Garcia-Vallvé S., Blay M. (2013). Grape Seed Procyanidin Extract Reduces the Endotoxic Effects Induced by Lipopolysaccharide in Rats. Free Radic. Biol. Med..

[45] Choi S.-K., Zhang X.-H., Seo J.-S. (2012). Suppression of Oxidative Stress by Grape Seed Supplementation in Rats. Nutr. Res. Pract..

[46] Berahmand F., Anoush G., Hosseini M.-J., Anoush M. (2020). Grape Seed Oil as a Natural Therapy in Male Rats with Alzheimer’s Diseases. Adv. Pharm. Bull..

[47] Rajput S.A., Sun L., Zhang N.-Y., Khalil M.M., Ling Z., Chong L., Wang S., Rajput I.R., Bloch D.M., Khan F.A. (2019). Grape Seed Proanthocyanidin Extract Alleviates AflatoxinB1-Induced Immunotoxicity and Oxidative Stress via Modulation of NF-κB and Nrf2 Signaling Pathways in Broilers. Toxins.

[48] Sheng K., Zhang G., Sun M., He S., Kong X., Wang J., Zhu F., Zha X., Wang Y. (2020). Grape Seed Proanthocyanidin Extract Ameliorates Dextran Sulfate Sodium-Induced Colitis through Intestinal Barrier Improvement, Oxidative Stress Reduction, and Inflammatory Cytokines and Gut Microbiota Modulation. Food Funct..

[49] Sharma S.D., Meeran S.M., Katiyar S.K. (2007). Dietary Grape Seed Proanthocyanidins Inhibit UVB-Induced Oxidative Stress and Activation of Mitogen-Activated Protein Kinases and Nuclear Factor-κB Signaling in *in Vivo* SKH-1 Hairless Mice. Mol. Cancer Ther..

[50] Jhun J.Y., Moon S.-J., Yoon B.Y., Byun J.K., Kim E.K., Yang E.J., Ju J.H., Hong Y.S., Min J.K., Park S.H. (2013). Grape Seed Proanthocyanidin Extract–Mediated Regulation of STAT3 Proteins Contributes to Treg Differentiation and Attenuates Inflammation in a Murine Model of Obesity-Associated Arthritis. PLoS ONE.

[51] Liu W., Ma Z., Kang J., Lin A., Wang Z., Chen H., Guo X., He X., Kang X. (2022). Grape Seed Proanthocyanidins Exert a Neuroprotective Effect by Regulating Microglial M1/M2 Polarisation in Rats with Spinal Cord Injury. Mediat. Inflamm..

[52] Busquets O., Carrasco M., Espinosa-Jiménez T., Ettcheto M., Verdaguer E., Auladell C., Bullò M., Camins A., Pinent M., Rodríguez-Gallego E. (2022). GSPE Pre-Treatment Protects against Long-Term Cafeteria Diet-Induced Mitochondrial and Inflammatory Affectations in the Hippocampus of Rats. Nutr. Neurosci..

[53] Rastegar-moghaddam S.H., Bigham M., Hosseini M., Ebrahimzadeh-bideskan A., Malvandi A.M., Mohammadipour A. (2022). Grape Seed Extract Effects on Hippocampal Neurogenesis, Synaptogenesis and Dark Neurons Production in Old Mice. Can This Extract Improve Learning and Memory in Aged Animals?. Nutr. Neurosci..

[54] Taghizadeh M., Malekian E., Memarzadeh M.R., Mohammadi A.A., Asemi Z. (2016). Grape Seed Extract Supplementation and the Effects on the Biomarkers of Oxidative Stress and Metabolic Profiles in Female Volleyball Players: A Randomized, Double-Blind, Placebo-Controlled Clinical Trial. Iran. Red Crescent Med. J..

[55] Argani H., Ghorbanihaghjo A., Vatankhahan H., Rashtchizadeh N., Raeisi S., Ilghami H. (2016). The Effect of Red Grape Seed Extract on Serum Paraoxonase Activity in Patients with Mild to Moderate Hyperlipidemia. Sao Paulo Med. J..

[56] De Groote D., Van Belleghem K., Devière J., Van Brussel W., Mukaneza A., Amininejad L. (2012). Effect of the Intake of Resveratrol, Resveratrol Phosphate, and Catechin-Rich Grape Seed Extract on Markers of Oxidative Stress and Gene Expression in Adult Obese Subjects. Ann. Nutr. Metab..

[57] Kar P., Laight D., Rooprai H.K., Shaw K.M., Cummings M. (2009). Effects of Grape Seed Extract in Type 2 Diabetic Subjects at High Cardiovascular Risk: A Double Blind Randomized Placebo Controlled Trial Examining Metabolic Markers, Vascular Tone, Inflammation, Oxidative Stress and Insulin Sensitivity. Diabet. Med..

[58] Turki K., Charradi K., Boukhalfa H., Belhaj M., Limam F., Aouani E. (2016). Grape Seed Powder Improves Renal Failure of Chronic Kidney Disease Patients. EXCLI J..

[59] Odai T., Terauchi M., Kato K., Hirose A., Miyasaka N. (2019). Effects of Grape Seed Proanthocyanidin Extract on Vascular Endothelial Function in Participants with Prehypertension: A Randomized, Double-Blind, Placebo-Controlled Study. Nutrients.

[60] Zhen L., Lange H., Crestini C. (2021). An Analytical Toolbox for Fast and Straightforward Structural Characterisation of Commercially Available Tannins. Molecules.

[61] Charradi K., Elkahoui S., Karkouch I., Limam F., Hassine F.B., Aouani E. (2012). Grape Seed and Skin Extract Prevents High-Fat Diet-Induced Brain Lipotoxicity in Rat. Neurochem. Res..

[62] Charradi K., Elkahoui S., Karkouch I., Limam F., Ben Hassine F., El May M.V., Aouani E. (2014). Protective Effect of Grape Seed and Skin Extract Against High-Fat Diet-Induced Liver Steatosis and Zinc Depletion in Rat. Dig. Dis. Sci..

[63] Vinson J.A., Proch J., Bose P. (2001). MegaNatural^®^ Gold Grapeseed Extract: In Vitro Antioxidant and In Vivo Human Supplementation Studies. J. Med. Food.

[64] Sies H. (2020). Oxidative Stress: Concept and Some Practical Aspects. Antioxidants.

[65] Deng G.-F., Shen C., Xu X.-R., Kuang R.-D., Guo Y.-J., Zeng L.-S., Gao L.-L., Lin X., Xie J.-F., Xia E.-Q. (2012). Potential of Fruit Wastes as Natural Resources of Bioactive Compounds. Int. J. Mol. Sci..

[66] Rinwa P., Kumar A. (2012). Piperine Potentiates the Protective Effects of Curcumin against Chronic Unpredictable Stress-Induced Cognitive Impairment and Oxidative Damage in Mice. Brain Res..

[67] Bagchi D., Bagchi M., Stohs S.J., Das D.K., Ray S.D., Kuszynski C.A., Joshi S.S., Pruess H.G. (2000). Free Radicals and Grape Seed Proanthocyanidin Extract: Importance in Human Health and Disease Prevention. Toxicology.

[68] Foshati S., Rouhani M.H., Amani R. (2021). The Effect of Grape Seed Extract Supplementation on Oxidative Stress and Inflammation: A Systematic Review and Meta-analysis of Controlled Trials. Int. J. Clin. Pract..

[69] Costa A.G.V., Garcia-Diaz D.F., Jimenez P., Silva P.I. (2013). Bioactive Compounds and Health Benefits of Exotic Tropical Red–Black Berries. J. Funct. Foods.

[70] Kulisic-Bilusic T., Schnäbele K., Schmöller I., Dragovic-Uzelac V., Krisko A., Dejanovic B., Milos M., Pifat G. (2009). Antioxidant Activity versus Cytotoxic and Nuclear Factor Kappa B Regulatory Activities on HT-29 Cells by Natural Fruit Juices. Eur. Food Res. Technol..

[71] Sung J., Lee J. (2010). Antioxidant and Antiproliferative Activities of Grape Seeds from Different Cultivars. Food Sci. Biotechnol..

[72] Pillay C.S., Eagling B.D., Driscoll S.R.E., Rohwer J.M. (2016). Quantitative Measures for Redox Signaling. Free Radic. Biol. Med..

[73] Jha J.C., Banal C., Chow B.S.M., Cooper M.E., Jandeleit-Dahm K. (2016). Diabetes and Kidney Disease: Role of Oxidative Stress. Antioxid. Redox Signal..

[74] Perrotta I., Aquila S. (2015). The Role of Oxidative Stress and Autophagy in Atherosclerosis. Oxidative Med. Cell. Longev..

[75] Leandro G.S., Sykora P., Bohr V.A. (2015). The Impact of Base Excision DNA Repair in Age-Related Neurodegenerative Diseases. Mutat. Res.-Fundam. Mol. Mech. Mutagen..

[76] Bolann B.J., Ulvik R.J. (1987). Release of Iron from Ferritin by Xanthine Oxidase. Role of the Superoxide Radical. Biochem. J..

[77] Habib H.M., El-Fakharany E.M., Kheadr E., Ibrahim W.H. (2022). Grape Seed Proanthocyanidin Extract Inhibits DNA and Protein Damage and Labile Iron, Enzyme, and Cancer Cell Activities. Sci. Rep..

[78] Vivanco I., Sawyers C.L. (2002). The Phosphatidylinositol 3-Kinase–AKT Pathway in Human Cancer. Nat. Rev. Cancer.

[79] Vivancos M., Moreno J.J. (2008). Effect of Resveratrol, Tyrosol and β-Sitosterol on Oxidised Low-Density Lipoprotein-Stimulated Oxidative Stress, Arachidonic Acid Release and Prostaglandin E_2_ Synthesis by RAW 264.7 Macrophages. Br. J. Nutr..

[80] Leifert W.R., Abeywardena M.Y. (2008). Grape Seed and Red Wine Polyphenol Extracts Inhibit Cellular Cholesterol Uptake, Cell Proliferation, and 5-Lipoxygenase Activity. Nutr. Res..

[81] Ben Nasr M., D’Addio F., Malvandi A.M., Faravelli S., Castillo-Leon E., Usuelli V., Rocchio F., Letizia T., El Essawy A.B., Assi E. (2018). Prostaglandin E2 Stimulates the Expansion of Regulatory Hematopoietic Stem and Progenitor Cells in Type 1 Diabetes. Front. Immunol..

[82] Zinovkin R.A., Grebenchikov O.A. (2020). Transcription Factor Nrf2 as a Potential Therapeutic Target for Prevention of Cytokine Storm in COVID-19 Patients. Biochem. Mosc..

[83] Scapagnini G., Sonya V., Nader A.G., Calogero C., Zella D., Fabio G. (2011). Modulation of Nrf2/ARE Pathway by Food Polyphenols: A Nutritional Neuroprotective Strategy for Cognitive and Neurodegenerative Disorders. Mol. Neurobiol..

[84] Surh Y.-J., Kundu J., Na H.-K. (2008). Nrf2 as a Master Redox Switch in Turning on the Cellular Signaling Involved in the Induction of Cytoprotective Genes by Some Chemopreventive Phytochemicals. Planta Med..

[85] Jain A., Lamark T., Sjøttem E., Bowitz Larsen K., Atesoh Awuh J., Øvervatn A., McMahon M., Hayes J.D., Johansen T. (2010). P62/SQSTM1 Is a Target Gene for Transcription Factor NRF2 and Creates a Positive Feedback Loop by Inducing Antioxidant Response Element-Driven Gene Transcription. J. Biol. Chem..

[86] Lu J., Jiang H., Liu B., Baiyun R., Li S., Lv Y., Li D., Qiao S., Tan X., Zhang Z. (2018). Grape Seed Procyanidin Extract Protects against Pb-Induced Lung Toxicity by Activating the AMPK/Nrf2/P62 Signaling Axis. Food Chem. Toxicol..

[87] Saba J., Couselo F.L., Bruno J., Carniglia L., Durand D., Lasaga M., Caruso C. (2022). Neuroinflammation in Huntington’s Disease: A Starring Role for Astrocyteand Microglia. Curr. Neuropharmacol..

[88] Huang G., Shi L.Z., Chi H. (2009). Regulation of JNK and P38 MAPK in the Immune System: Signal Integration, Propagation and Termination. Cytokine.

[89] Churchward M.A., Michaud E.R., Mullish B.H., Miguens Blanco J., Garcia Perez I., Marchesi J.R., Xu H., Kao D., Todd K.G. (2023). Short-Chain Fatty and Carboxylic Acid Changes Associated with Fecal Microbiota Transplant Communally Influence Microglial Inflammation. Heliyon.

[90] Baron G., Altomare A., Della Vedova L., Gado F., Quagliano O., Casati S., Tosi N., Bresciani L., Del Rio D., Roda G. (2024). Unraveling the Parahormetic Mechanism Underlying the Health-Protecting Effects of Grapeseed Procyanidins. Redox Biol..

[91] Perez-Pardo P., Dodiya H.B., Engen P.A., Forsyth C.B., Huschens A.M., Shaikh M., Voigt R.M., Naqib A., Green S.J., Kordower J.H. (2019). Role of TLR4 in the Gut-Brain Axis in Parkinson’s Disease: A Translational Study from Men to Mice. Gut.

[92] Valadão P.A.C., Santos K.B.S., Ferreira E Vieira T.H., Macedo E Cordeiro T., Teixeira A.L., Guatimosim C., De Miranda A.S. (2020). Inflammation in Huntington’s Disease: A Few New Twists on an Old Tale. J. Neuroimmunol..

[93] Janle E.M., Lila M.A., Grannan M., Wood L., Higgins A., Yousef G.G., Rogers R.B., Kim H., Jackson G.S., Ho L. (2010). Pharmacokinetics and Tissue Distribution of^14^ C-Labeled Grape Polyphenols in the Periphery and the Central Nervous System Following Oral Administration. J. Med. Food.

[94] Mabrouk M., El Ayed M., Démosthènes A., Aissouni Y., Aouani E., Daulhac-Terrail L., Mokni M., Bégou M. (2022). Antioxidant Effect of Grape Seed Extract Corrects Experimental Autoimmune Encephalomyelitis Behavioral Dysfunctions, Demyelination, and Glial Activation. Front. Immunol..

[95] Jiang M., Wang J., Fu J., Du L., Jeong H., West T., Xiang L., Peng Q., Hou Z., Cai H. (2012). Neuroprotective Role of Sirt1 in Mammalian Models of Huntington’s Disease through Activation of Multiple Sirt1 Targets. Nat. Med..

[96] Prasain J.K., Peng N., Dai Y., Moore R., Arabshahi A., Wilson L., Barnes S., Michael Wyss J., Kim H., Watts R.L. (2009). Liquid Chromatography Tandem Mass Spectrometry Identification of Proanthocyanidins in Rat Plasma after Oral Administration of Grape Seed Extract. Phytomedicine.

[97] Sun Q., Jia N., Li X., Yang J., Chen G. (2019). Grape Seed Proanthocyanidins Ameliorate Neuronal Oxidative Damage by Inhibiting GSK-3β-Dependent Mitochondrial Permeability Transition Pore Opening in an Experimental Model of Sporadic Alzheimer’s Disease. Aging.

[98] Endlicher R., Drahota Z., Štefková K., Červinková Z., Kučera O. (2023). The Mitochondrial Permeability Transition Pore—Current Knowledge of Its Structure, Function, and Regulation, and Optimized Methods for Evaluating Its Functional State. Cells.

[99] Fu K., Chen L., Miao L., Guo Y., Zhang W., Bai Y. (2019). Grape Seed Proanthocyanidins Protect N2a Cells against Ischemic Injury via Endoplasmic Reticulum Stress and Mitochondrial-Associated Pathways. CNS Neurol. Disord.-Drug Targets.

[100] Shanmughapriya S., Rajan S., Hoffman N.E., Zhang X., Guo S., Kolesar J.E., Hines K.J., Ragheb J., Jog N.R., Caricchio R. (2015). Ca^2+^ Signals Regulate Mitochondrial Metabolism by Stimulating CREB-Mediated Expression of the Mitochondrial Ca^2+^ Uniporter Gene *MCU*. Sci. Signal..

[101] Zhao S., Zhang L., Yang C., Li Z., Rong S. (2019). Procyanidins and Alzheimer’s Disease. Mol. Neurobiol..

[102] Arabit J., Elhaj R., Schriner S., Sevrioukov E., Jafari M. (2018). Rhodiola rosea improves lifespan, locomotion, and neurodegeneration in a Drosophila melanogaster model of Huntington’s disease. Biomed. Res. Int..

[103] García-Aguilar A., Palomino O., Benito M., Guillén C. (2021). Dietary Polyphenols in Metabolic and Neurodegenerative Diseases: Molecular Targets in Autophagy and Biological Effects. Antioxidants.

[104] Nabavi S.F., Sureda A., Dehpour A.R., Shirooie S., Silva A.S., Devi K.P., Ahmed T., Ishaq N., Hashim R., Sobarzo-Sánchez E. (2018). Regulation of Autophagy by Polyphenols: Paving the Road for Treatment of Neurodegeneration. Biotechnol. Adv..

[105] Capiralla H., Vingtdeux V., Zhao H., Sankowski R., Al-Abed Y., Davies P., Marambaud P. (2012). Resveratrol Mitigates Lipopolysaccharide- and Aβ-mediated Microglial Inflammation by Inhibiting the TLR4/NF-κB/STAT Signaling Cascade. J. Neurochem..

[106] Hwang S.H., Shin E.-J., Shin T.-J., Lee B.-H., Choi S.-H., Kang J., Kim H.-J., Kwon S.-H., Jang C.-G., Lee J.-H. (2012). Gintonin, a Ginseng-Derived Lysophosphatidic Acid Receptor Ligand, Attenuates Alzheimer’s Disease-Related Neuropathies: Involvement of Non-Amyloidogenic Processing. J. Alzheimer’s Dis..

[107] Kanellos P.T., Kaliora A.C., Tentolouris N.K., Argiana V., Perrea D., Kalogeropoulos N., Kountouri A.M., Karathanos V.T. (2014). A Pilot, Randomized Controlled Trial to Examine the Health Outcomes of Raisin Consumption in Patients with Diabetes. Nutrition.

[108] Chuang C.-C., Bumrungpert A., Kennedy A., Overman A., West T., Dawson B., McIntosh M.K. (2011). Grape Powder Extract Attenuates Tumor Necrosis Factor α-Mediated Inflammation and Insulin Resistance in Primary Cultures of Human Adipocytes. J. Nutr. Biochem..

[109] Kim H., Kim J.Y., Song H.S., Park K.U., Mun K.-C., Ha E. (2011). Grape Seed Proanthocyanidin Extract Inhibits Interleukin-17-Induced Interleukin-6 Production via MAPK Pathway in Human Pulmonary Epithelial Cells. Naunyn-Schmiedeberg’s Arch. Pharmacol..

[110] Liperoti R., Vetrano D.L., Bernabei R., Onder G. (2017). Herbal Medications in Cardiovascular Medicine. J. Am. Coll. Cardiol..

[111] Abedini S., Pourghassem B., Babaei H., Aliasgarzadeh A., Poorabdollahi P. (2013). Effect of Supplementation with Grape Seed Extract (Vitis Vinifera) on Serum Lipid Profiles in Patient with Type 2 Diabetes. Iran. J. Endocrinol. Metab..

[112] Mellen P.B., Daniel K.R., Brosnihan K.B., Hansen K.J., Herrington D.M. (2010). Effect of Muscadine Grape Seed Supplementation on Vascular Function in Subjects with or at Risk for Cardiovascular Disease: A Randomized Crossover Trial. J. Am. Coll. Nutr..

[113] Park E., Edirisinghe I., Choy Y.Y., Waterhouse A., Burton-Freeman B. (2016). Effects of Grape Seed Extract Beverage on Blood Pressure and Metabolic Indices in Individuals with Pre-Hypertension: A Randomised, Double-Blinded, Two-Arm, Parallel, Placebo-Controlled Trial. Br. J. Nutr..

[114] Pourghassem Gargari B., Abedini S., Babaei H., Aliasgarzadeh A., Pourabdollahi P. (2011). Effect of Supplementation with Grape Seed (Vitis Vinifera) Extract on Antioxidant Status and Lipid Peroxidation in Patient with Type ΙΙ Diabetes. J. Med. Plants Res..

[115] Sano A., Uchida R., Saito M., Shioya N., Komori Y., Tho Y., Hashizume N. (2007). Beneficial Effects of Grape Seed Extract on Malondialdehyde-Modified LDL. J. Nutr. Sci. Vitaminol..

[116] Sano A. (2017). Safety Assessment of 4-Week Oral Intake of Proanthocyanidin-Rich Grape Seed Extract in Healthy Subjects. Food Chem. Toxicol..

[117] Arola-Arnal A., Oms-Oliu G., Crescenti A., Del Bas J.M., Ras M.R., Arola L., Caimari A. (2013). Distribution of Grape Seed Flavanols and Their Metabolites in Pregnant Rats and Their Fetuses. Mol. Nutr. Food Res..

[118] Zielinsky P., Piccoli A.L., Manica J.L., Nicoloso L.H., Menezes H., Busato A., Moraes M.R., Silva J., Bender L., Pizzato P. (2010). Maternal Consumption of Polyphenol-Rich Foods in Late Pregnancy and Fetal Ductus Arteriosus Flow Dynamics. J. Perinatol..

[119] Etheridge A., Black S., Patel P., So J., Mathews J. (2007). An in Vitro Evaluation of Cytochrome P450 Inhibition and P-Glycoprotein Interaction with Goldenseal, *Ginkgo Biloba*, Grape Seed, Milk Thistle, and Ginseng Extracts and Their Constituents. Planta Med..

[120] Wanwimolruk S., Wong K., Wanwimolruk P. (2009). Variable Inhibitory Effect of Different Brands of Commercial Herbal Supplements on Human Cytochrome P-450 CYP3A4. Drug Metab. Drug Interact..

